# ProG-SOL: Predicting Protein Solubility Using Protein Embeddings and Dual-Graph Convolutional Networks

**DOI:** 10.1021/acsomega.4c09688

**Published:** 2025-01-24

**Authors:** Gen Li, Ning Zhang, Long Fan

**Affiliations:** † Production and R&D Center I of LSS, GenScript (Shanghai) Biotech Co., Ltd., Shanghai 200131, China; ‡ Production and R&D Center I of LSS, GenScript Biotech Corporation, Nanjing 211122, China

## Abstract

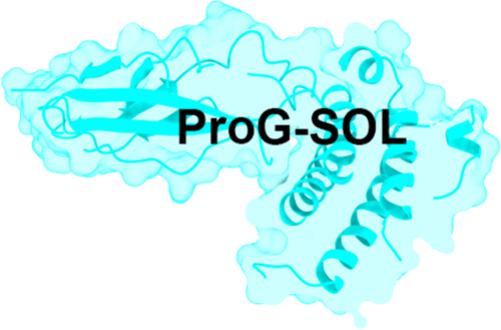

Solubility is a key biophysical property of proteins and is essential for evaluating the effectiveness of proteins in biochemical engineering. In recent years, the prediction method of protein solubility has received extensive attention in the protein engineering research community. Many methods have been developed to predict protein solubility, but the generalization performance of existing prediction methods on independent test sets must be improved. In addition, solubility prediction methods do not work well when they are used for regression tasks. To address these issues, we developed a new method, ProG-SOL, an innovative sequence-based dual-graph convolutional network that simultaneously exploits the protein pretrained graph and the protein evolutionary graph for assessing solubility. Compared with other methods, ProG-SOL achieves better classification and regression results for different independent test sets at the same time. The model framework of our method may also be used to predict other properties of proteins such as protein function, protein–protein interaction, protein folding, and drug design, which provide broad application prospects in protein engineering.

## 1 Introduction

Solubility is an important biophysical property for evaluating the effectiveness of proteins in biological and chemical engineering.^1^ The solubility of proteins is the result of the interaction between various external physical conditions (such as pH and temperature) and intrinsic factors (such as the protein’s primary sequence and tertiary structure).^2^ However, a common problem during protein production is the formation of inclusion bodies in host cells. Inclusion bodies are aggregates of nonfunctional, improperly folded proteins, which are usually insoluble or with very low solubility.^3^ Heterologous expression of proteins in model host cells (such as *Escherichia coli*) usually results in the formation of inclusion bodies.^4^ The formation of inclusion bodies reduces the solubility of proteins and poses challenges to the subsequent purification and functional studies of proteins. In early studies, remedies such as using weak promoters, strong denaturants, refolding,^5^ low-temperature expression,^6^ or codon optimization^7^ were commonly used to improve the solubility of target proteins. Nevertheless, in the context of the explosive growth of available protein sequences, some prediction methods need to be developed for reducing the time and cost of wet experiments, which will help to refine candidate proteins to those with a high probability of solubility.

In recent years, the prediction technology of protein solubility has received considerable attention in the protein engineering research community. Many methods for predicting protein solubility have been developed, which can be roughly divided into three categories according to the algorithm: (i) statistical-based methods, such as SWI;^8^ (ii) traditional machine learning-based methods, such as CamSol,^9^ Protein-Sol,^10^ PaRSnIP,^11^ and SoluProt;^12^ (iii) deep learning-based methods, such as DeepSol,^13^ EPSOL,^14^ NetSolP,^15^ GraphSol,^16^ HybridGCN,^1^ and DeepSoluE.^17^ Despite the achievements, the generalization performance of existing prediction methods in different independent test sets must be improved. In addition, solubility prediction methods perform poorly when they are used for regression prediction. This is important for applications that require knowledge of the exact solubility level. The ability of Natural Language Processing (NLP) to recover information with context dependency is highly suitable for application in biological sequences, especially protein sequences.^18^ The groundbreaking advancements in NLP have a notable impact on protein research, promoting the application of language model concepts to protein sequences in various studies, such as UniRep,^19^ EMS-1b,^20^ TAPE,^21^ and ProtTrans.^22^ These studies reveal that protein sequence embeddings (vector representations produced by pretrained models) are highly accurate in predicting SCOP^6^ and protein properties.^1,23,24^ These pretrained models offer a unique perspective on the language of proteins through embeddings, proving to be highly effective in addressing a variety of downstream tasks and showing significant improvements over earlier supervised machine learning methods.

Inspired by previous works, we presented ProG-SOL, an innovative sequence-based dual-graph convolutional network that simultaneously exploits the protein pretrained graph and the protein evolutionary graph to provide a method for assessing solubility. ProG-SOL combines the predicted contact probabilities with protein embedding and PSSM features as inputs to two different SAGE convolutional blocks to train the model. A protein graph is used to capture short-range residue interactions, and a language model is used to represent long-range protein sequence information. The two graphs provide pseudostructural information and evolutionary distance information on residues, respectively. In addition, the global protein embedding, evolutionary information, and residue interaction relationships enhance the feature representation ability of our method. These features provide a wealth of information about protein function and structure, thereby substantially improving the model’s accuracy in predicting protein solubility. Our method is characterized by the establishment of a prediction model that is compatible with both classification and regression tasks. It achieved better generalization performance than other methods on a series of independent test sets and possessed broad application prospects.

## 2 Materials and Methods

### 2.1 Data Set for Model Development

#### 2.1.1 PSI: Biology Data Set

For the model training, we used the “PSI: Biology” data set with 11,226 protein samples obtained from a previous study^15^ as the training set to build a binary classification model. After removing samples with conflicting labels for the same protein sequence, the final data set used to train the model contained 11,110 samples. According to the classification criteria of Smialowski et al.,^25^ the number of samples of soluble proteins and insoluble proteins was 7402 and 3708, respectively.

### 2.2 Independent Data Sets for Model Testing

In order to test the generalization performance of our model, we used the following four independent test sets to validate the model and deleted all samples in the test set with a global protein sequence identity higher than 25% with the training set. The global sequence similarity alignment tool used 32-bit USEARCH v11.0.667.^26^

#### 2.2.1 NESG Data Set

The North East Structural Consortium (NESG) expressed 1,323 proteins in *E. coli* using a unified production process^27^ and Hon et al. scored their solubility.^12^ We used these data as an independent data set and compared them with the training set for global sequence alignment. After screening, 1319 samples were retained, of which 838 were soluble proteins and 481 were insoluble proteins.

#### 2.2.2 eSOL Data Set

Niwa et al.^28^ synthesized the entire *E. coli* protein using an in vitro reconstitution translation system and analyzed the aggregation tendency of the protein, and finally successfully quantified 3173 proteins. According to the author’s definition, solubility is the ratio of the supernatant fraction to the total fraction in physiochemical experiments referred to as PURE, and thresholds of 30 and 70% are used to define insoluble and soluble proteins. The eSOL data set was globally aligned with the training set, and 2943 proteins were retained after filtering for regression tasks. According to the solubility threshold defined by the original author, we obtained 2067 proteins for the application of classification tasks, of which the number of samples of soluble proteins and insoluble proteins were 928 and 1139, respectively.

#### 2.2.3 *Saccharomyces cerevisiae* Data Set

This data set is collected from the work of Hou et al.^29^ and contains 109 protein samples expressed in *Saccharomyces cerevisiae*. Under the same external conditions, in order to reduce the impact of the environment, the solubility was also measured by a cell-free expression system called PURE. After global sequence alignment and filtering of the data set with the training set, 103 samples were retained for regression problem testing. We classified the data set using the same solubility threshold as the eSOL data set and finally obtained 72 samples for classification task testing, including 53 soluble proteins and 19 insoluble proteins.

#### 2.2.4 DMS Data Sets

Klesmith et al.^30^ performed deep mutation scanning (DMS) using yeast surface display (YSD) screening to measure the solubility of TEM-1 β-lactamase variant (TEM-1.1) and levoglucosan kinase (LGK). The number of single-point mutation data with solubility scores for TEM-1.1 in YSD screening is 4126 (632 mutations increased solubility and 3494 mutations decreased solubility), while the number of single-point mutation data with solubility scores for LGK in YSD screening is 6298 (314 mutations increased solubility and 5984 mutations decreased solubility). For this data set, we utilized both the protein sequences before and after mutation to predict their respective solubility probabilities. By calculating the solubility probabilities for both the wild-type and mutant sequences, we derived the solubility change upon mutation by subtraction. This approach allows us to evaluate our method’s performance on mutation-related problems, which has also been adopted by other methods.

The eSOL and *S. cerevisiae* data sets contain the amount of soluble proteins in solution and divide them into soluble and insoluble proteins according to the proportion of soluble proteins. Therefore, these two data sets can be used for classification and regression tasks (calculate PCC with predicted solubility scores). Other data sets are used for classification tasks because they contain only classification labels.

### 2.3 GraphSAGE Convolution Network Architecture

Graph is the most basic part of GraphSAGE, which consists of two parts: nodes (vertices) and edges. Specifically, given a graph G = (V, E, W), where V is the set of K nodes and E is the edge. In addition, each edge can be assigned a weight of W. The input of GraphSAGE is divided into two parts. The first part is the node features of dimension L × F, where L is the number of nodes and F is the dimension of features for each node. The second part is the adjacency matrix of dimensions L × L. In a protein, nodes represent the amino acids that make up the protein, and the residue interactions constitute the adjacency matrix. In this work, we use the class of dgl.nn.pytorch.conv.SAGEConv in DGL (version 2.1.0)^31^ to develop a ProG-SOL model based on GraphSAGE.

#### 2.3.1 Nodes of Pretrained Graph

In this work, we employ protein sequence embeddings generated by the ProtT5-XL-Uniref50 pretrained model as node features. The ProtT5-XL-Uniref50 model developed by Elnaggar et al.^32^ was trained on hundreds of millions of protein sequences using NLP transformers, thereby capturing a range of amino acid’s properties related to protein structure and function. This model achieves state-of-the-art performance across various downstream tasks compared to other popular protein language models.^33^ When a protein sequence is input into the ProtT5-XL-Uniref50 pretrained model, the model encodes each amino acid in the sequence using an encoder. Each amino acid is represented as a vector of length 1024, resulting in a vector of dimensions L × 1024 for a protein sequence of length L. The model consists of a 24-layer architecture. To maximize the information regarding protein solubility, we chose output from the final layer.

#### 2.3.2 Nodes of Evolutionary Graph

Evolutionary features used in this method were generated by 3 iterations of PSI-BLAST v2.4.0^34^ against the UniRef90 protein sequence database.^35^ Specifically, a protein sequence of length L is processed to produce a position-specific scoring matrix (PSSM) profile. This PSSM profile is then used to create an evolutionary feature matrix of size L × 20, where each position in the protein sequence is represented by a vector of 20 scores corresponding to the likelihood of each standard amino acid.

#### 2.3.3 Edges and Its Weight

To avoid using the protein structure, we exploited SPOT-Contact-LM^36^ to predict residue interaction networks from protein sequences, and set the outputting probabilities as edge weights, while edges were assumed to be fully connected. Therefore, the edge features and weight matrix of a protein contact graph of length L is an L × L vector.

#### 2.3.4 Training Details

The specific parameters in the training process are set as follows: The three-layer graph convolution network for the pretrained graph has an output dimension of 128 for each layer, while the two-layer graph convolution network for the evolutionary graph has an output dimension of 32 for each layer. The fully connected layer consists of 128 and 2 neurons. The loss function is a binary cross-entropy loss. Optimization is performed using the Adam optimizer with hyperparameters β1 = 0.9, β2 = 0.99, and ε = 1 × 10^–8^, and a learning rate of 0.01. An L2 regularization coefficient of 1 × 10^–3^ is applied. A dropout rate of 0.3 is used in the graph convolution layers, and neighbor node information is aggregated using average pooling. The ReLU activation function is used throughout the training process, and the output layer employs a softmax function for binary classification. The batch size is 512, and the training is conducted over 30 epochs.

### 2.4 Evaluation Metrics

Our method uses a threshold of 0.5 to classify protein solubility; that is, if the predicted soluble probability of a protein is higher than 0.5, it is classified as a soluble protein; otherwise, it is an insoluble protein. We used a variety of classification metrics to evaluate the predictive performance of the model, including the area under curve (AUC), accuracy (ACC), F1 score, and Pearson’s correlation coefficient (PCC), which are defined as follows:
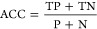

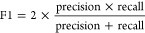
Among them, TP, FP, TN, and FN represent true positive, false positive, true negative, and false negative, respectively. By the way, PCC is used only to evaluate the regression task (eSOL and *S. cerevisiae* data sets).

## 3 Result

### 3.1 Designing a Dual-Graph Convolutional Network for Solubility Prediction

**Figure 1:**
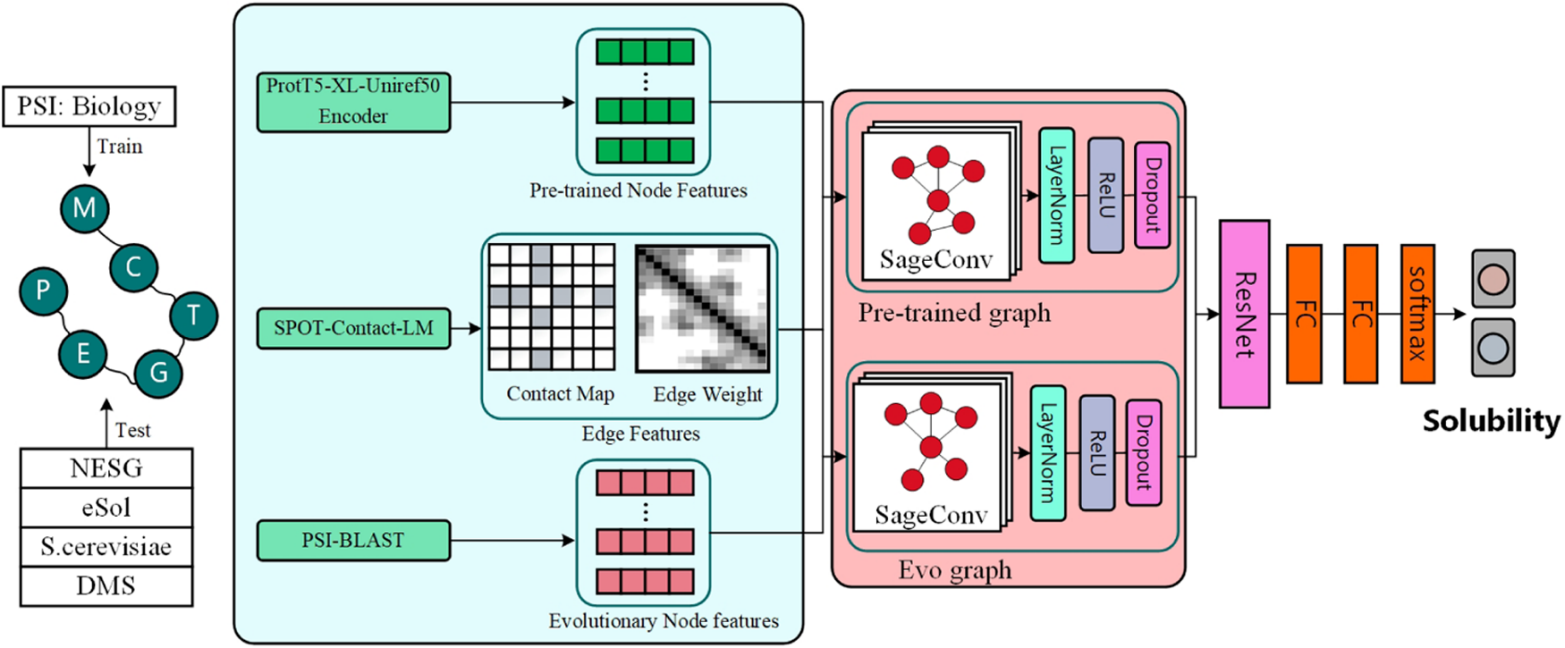
Development schematic of the ProG-SOL model architectures. ProG-SOL is a dual GraphSAGE architecture, where ProtT5-XL-Uniref50 embeddings and PSSM provide node features. Edges and weights are defined by SPOT-Contact-LM outputting. A graph convolutional layer takes in both the contact map and the embedding from the previous layer and then outputs the embedding in the next layer. ResNet concatenates the embeddings of the two graph convolutional layers into a matrix. We then use two fully connected layers to compute the embedding from the residual network representation. Finally, the binary classification is predicted by the softmax.

Protein solubility prediction is a classification task, and the goal is to predict the probability of its solubility by putting it in a protein sequence. In this work, we proposed a deep framework based on GraphSAGE, called ProG-SOL, for protein solubility prediction. As shown in Figure 1, the architecture of the ProG-SOL model consists of three modules: (1) Features exaction. To represent protein sequences with graphs, we use the protein embedding extracted from the last hidden layer of ProtT5-XL-Uniref50 and the evolutionary information obtained from PSSM as two types of node features (V), which contain the general and evolutionary characteristics of the target protein sequence, respectively. Due to the vast size of our training set, we do not derive edges and weights from the experimental or predicted structures. Instead, we use the protein contact map and its probability, as predicted by SPOT-Contact-LM,^36^ to define the edges (E) and weights (W). It should be noted that in order to obtain as much information about the edges as possible, we regard all the edges as connections. (2) Graph networks. We design a dual-graph network to utilize the information on different node features. (i) pretrained graph, which combines the sequence embedding extracted from the pretrained model with the predicted contact probabilities matrix as input. The node features are updated through concatenation and aggregation after passing through three GraphSAGE layers. (ii) Evolutionary graph, which combines the PSSM feature matrix and the predicted contact probabilities matrix as input and updates the node features through concatenation and aggregation after two GraphSAGE layers. In both the physical and evolutionary graphs, we apply a dropout layer to the node features to prevent overfitting. (3) Multilayer perceptron (MLP). The features obtained from the pretrained graph and the evolutionary graph are combined to represent the feature representation of the entire protein sequence. The resulting feature representation is then fed into a fully connected layer with the ReLU function employed as the activation function. The output layer uses the softmax function for binary classification.

### 3.2 5-fold Cross-Validation

**Figure 2:**
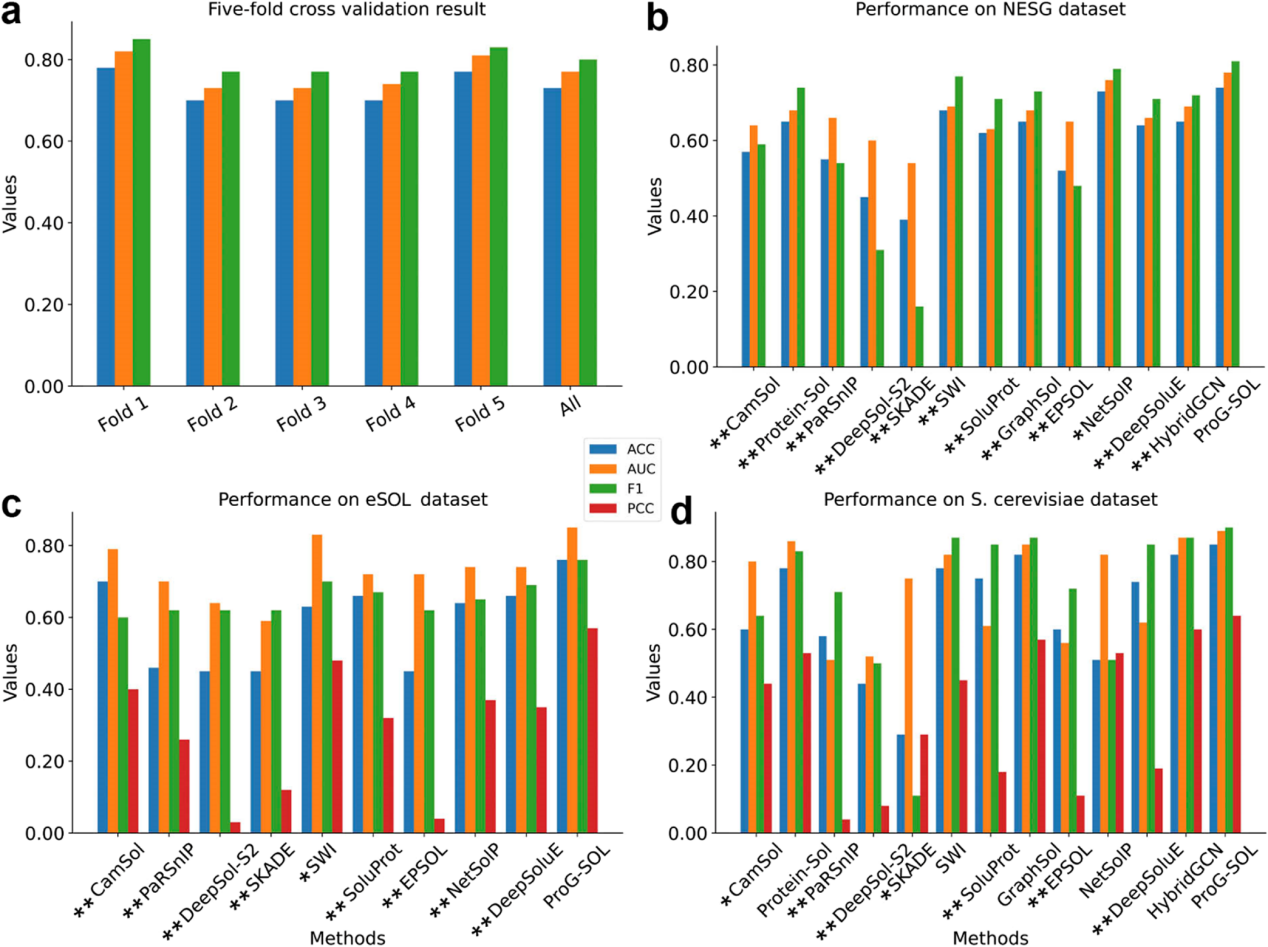
Comparison of ProG-SOL with other predictors on different data sets. (a) 5-fold cross-validation result on the training data set. Independent test on (b) NESG (c) eSOL (d) *S. cerevisiae* data set. “**” indicates a significant difference (AUC, *p* < 0.01), and “*” indicates a difference (AUC, *p* < 0.05), tested by DeLong’s test. Detailed metrics can be found in Tables S1–S3.

We trained the ProG-SOL model on the PSI: Biology data set as described in the Section 2. We evaluated the robustness and generalizability of the ProG-SOL on the PSI:Biology data set using a carefully curated 5-fold cross-validation regarding previous studies,^15^ i.e., a balanced partitioning of the labels to ensure that each training and test data set did not share sequences with global sequence identity >25%. As shown in Figure 2a, the accuracy and the AUC values of the ProG-SOL model for the 5-fold cross-validation are 0.73 and 0.77, respectively, which proves the generalization and fitting capabilities.

### 3.3 ProG-SOL Outperforms Other SOTA Methods on Different Independent Data Sets

ProG-SOL is a novel tool designed to predict the protein solubility. An essential step for evaluating the developed model involves independent testing, which allows for comparisons with other solubility predicted approaches, including machine learning models, deep learning models, and empirical models. In order to compare our method with that of predictors, we collected four independent test sets. These results show that our method achieves better generalization performance than other methods on three independent test sets and is compatible with both classification and regression tasks.

#### 3.3.1 Independent Test on Two Data Sets: NESG and eSOL

The first test data set, introduced by Hon et al.,^12^ comprises 9644 proteins expressed in *E. coli*. The data set features integer values ranging from 0 to 5, intended for use in classification tasks. The results of different methods on the NESG data set are presented in Figure 2b, from which we can find ProG-SOL achieves the best ACC (0.74) and AUC (0.78) among the comparison methods.

eSOL is another renowned and widely utilized solubility data set^28^ that includes experimentally determined solubility for more than 3000 *E. coli* proteins produced using the PURE cell-free expression system.^37^ eSOL is notable for its extensive collection of highly consistent data, making it a valuable resource for precise solubility information. The data were generated through a rigorous process to provide an accurate quantitative measure of thermodynamic solubility. This data set evaluates the classification performance of the predictors and the regression performance of the model, which reflects the quantitative ability of the model to a certain extent. The performances on eSOL data set are reported in Figure 2c, in which the performance of ProG-SOL with ACC (0.76) and AUC (0.85) is significantly higher than that of other methods. At the same time, when applying ProG-SOL to the regression task on eSOL data set, the Pearson’s correlation coefficient (PCC) reached 0.57, showing our method is compatible with both classification and regression tasks.

#### 3.3.2 Comparisons with SOTA Methods on the *S. cerevisiae* Data Set

We also compared our ProG-SOL with other SOTA performance methods on the *S. cerevisiae* data set, which includes 109 protein samples expressed in *S. cerevisiae*. After aligning and filtering, 103 samples remained for regression testing. Using the eSOL solubility threshold, 72 samples were classified: 53 soluble and 19 insoluble proteins. As for the result, ProG-SOL also achieved the best performance, with an ACC of 0.85, an AUC of 0.90, and a PCC of 0.64 (Figure 2d).

### 3.4 Performance on DMS Data Sets for TEM-1 β-Lactamase Variant and Levoglucosan Kinase

**Figure 3:**
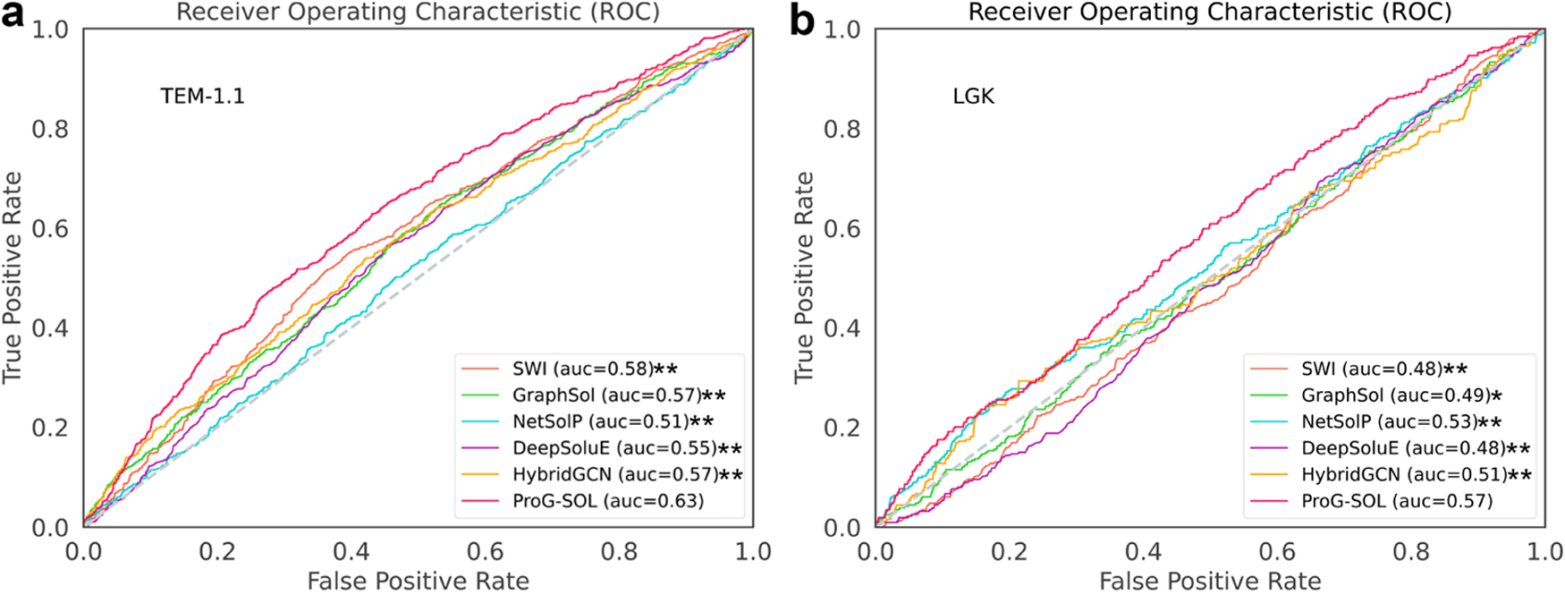
Performance of prediction methods on DMS data sets for TEM-1.1 and LGK. (a, b) show the receiver operating characteristic (ROC) curves of all methods for YSD screening on the TEM-1.1 and LGK test sets. “**” indicates a significant difference (*p* < 0.01), and “*” indicates a difference (*p* < 0.05), tested by DeLong’s test.

DMS data set is the third independent data, which is composed of 8,099 solubility scores determined by DMS (Deep mutational scanning) technology.^38^ Klesmith et al.^30^ performed DMS using yeast surface display (YSD) screening to measure the solubility of TEM-1 β-lactamase variant (TEM-1.1) and levoglucosan kinase (LGK). The sequence identities of TEM-1.1 and LGK share less than 25% with our training set. Figure 3 displays a comparison of AUC obtained by using 5 SOTA predictors. (Only the top 5 methods ranked in NESG and eSOL were selected). ProG-SOL outperforms all other predictors in terms of AUC, with a value of 0.63 and 0.57 for TEM-1.1 and LGK, respectively. Although our method does not show superior results, it still has significant differences compared to other methods. Furthermore, it has been demonstrated that our predicted structures can be utilized for solubility assessments without relying on experimental structures. This significantly enhances the practicality and accessibility of our method.

### 3.5 Ablation Experiment of ProG-SOL

ProG-SOL is composed of various modules sourced from multiple origins. To determine the significance of each component, we conducted ablation studies by removing key elements from the well-established ProG-SOL framework. Table 1 illustrates the validation performance of these modified ProG-SOL variants in predicting protein solubility, underscoring the necessity of all components for the model’s final performance. Specifically, individual graphs were utilized instead of dual graphs, and contact graphs were processed with different thresholds (Tables S4 and S5). Additionally, we evaluated the performance of ProG-SOL across all data sets using GraphConv, SAGEConv, GINConv, GATConv, and ChebConv, with GraphConv emerging as the top performer. We used the same parameters for 10 training runs, and the results showed that the maximum standard error on all test sets did not exceed 0.02 (Table S6). This methodology led to significant enhancements in both prediction accuracy and model stability.

**Table 1:** Ablation Study of ProG-SOL^a^

	5-fold cross-validation			NESG			eSOL			*S. cerevisiae*		
modules	ACC	AUC	F1	ACC	AUC	F1	ACC	AUC	F1	ACC	AUC	F1
pretrained graph	0.73	0.73	0.80	0.71	0.77	0.80	0.80	0.81	0.64	0.83	0.85	0.90
evo graph	0.70	0.64	0.80	0.70	0.70	0.80	0.52	0.80	0.64	0.78	0.74	0.87
single graph	0.73	0.75	0.80	0.72	0.77	0.81	0.51	0.82	0.64	0.83	0.85	0.90
dual-graph (ProG-SOL)	**0.73**	**0.77**	**0.80**	**0.74**	**0.78**	**0.81**	**0.76**	**0.85**	**0.75**	**0.85**	**0.89**	**0.90**

a Pretrained graph: Only use the protein embedding graph as the input. Evo graph: Only use the evolutionary graph as the input. Single graph: Use a single graph whose node features are concatenated from protein embedding and evolutionary information. The best results are in bold.

## 4 Discussion

Protein solubility is crucial in many fields, including biochemistry, pharmaceuticals, and biotechnology, as it affects the production, formulation, and effectiveness of protein-based products. By the development of a prediction method, researchers can save time and resources by identifying potentially soluble proteins before conducting laboratory experiments. This can accelerate the development of new drugs, enzymes, and other protein-based products. In this study, we proposed a new GraphSAGE convolutional network, ProG-SOL, which combines protein embedding, classical evolutionary information, and contact maps to predict the solubility of a given protein. Unlike most predictors that merge all features into one graph, we combine protein embeddings, evolutionary features, and predicted contact probabilities into two different convolutional graphs. This structure is conducive to capturing additional information useful for solubility prediction so that the model can achieve better predictive performance and help ProG-SOL outperform other predictors on various blind test sets, especially in terms of accuracy and AUC. We believe that our approach will contribute to advancing the application of graph neural networks in prediction for protein property tasks.

## Data Availability

All the data sets and analysis code can be found in https://github.com/GenScript-IBDPE/ProG-SOL

## Supporting Information Available

Detailed metrics of prediction methods on NESG, eSol and *S. cerevisiae* data set (Tables S1–S3). Ablation study of ProG-SOL on different graph network models (Table S4) and different predicted contact probability cutoffs (Table S5). ProG-SOL model robustness test (Table S6). Pseudocode of ProG-SOL. (PDF)
