# Supplementary material for: ProG-SOL: Predicting Protein Solubility Using Protein Embeddings and Dual-Graph Convolutional Networks
Source: ACS Omega. 2025 Jan 24;10(4):3910–6. doi: 10.1021/acsomega.4c09688 (PMC11800053; doi:10.1021/acsomega.4c09688)
Supplement: Supplementary file 1 — ao4c09688_si_001.pdf [file ao4c09688_si_001.pdf]

ProG-SOL: Predicting Protein Solubility Using Protein Embeddings and dual-Graph Convolutional Networks

Gen Li[a]†, *Ning Zhang*[b]† *and Long Fan*[a*]

[a]Production and R&D Center I of LSS, GenScript (Shanghai) Biotech Co.,Ltd., Shanghai, 200131, China

[b]Production and R&D Center I of LSS, GenScript Biotech Corporation, Nanjing, 211122, China

†These authors contributed equally to this work.

*To whom correspondence should be addressed. Email: leo.fan@genscript.com

Table S1. Performance of prediction methods on NESG dataset.

Methods	ACC	AUC	MCC	F1	Precision	Recall
CamSol	0.568	0.644	0.196	0.586	0.749	0.481
Protein-Sol	0.647	0.678	0.208	0.736	0.700	0.777
PaRSnIP	0.553	0.661	0.212	0.542	0.777	0.416
DeepSol-S2	0.451	0.596	0.118	0.313	0.764	0.197
SKADE	0.393	0.543	0.016	0.159	0.661	0.090
SWI	0.679	0.689	0.268	0.767	0.711	0.832
SoluProt	0.624	0.634	0.187	0.705	0.703	0.706
GraphSol	0.650	0.682	0.234	0.729	0.717	0.742
EPSOL	0.518	0.650	0.171	0.479	0.764	0.348
NetSolP	0.731	0.760	0.411	0.793	0.777	0.809
DeepSoluE	0.637	0.658	0.224	0.711	0.720	0.703
HybridGCN	0.654	0.692	0.273	0.717	0.745	0.691
ProG-SOL	0.743	0.780	0.424	0.811	0.761	0.868

Note: the predictions of DeepSoluE on NESG dataset contain 7 NaN values.

Table S2. Performance of prediction methods on eSol dataset.

Methods	ACC	AUC	MCC	F1	Precision	Recall	PCC
CamSol	0.701	0.787	0.399	0.595	0.761	0.488	0.397
PaRSnIP	0.456	0.701	0.023	0.617	0.451	0.975	0.263
DeepSol-S2	0.450	0.641	0.028	0.620	0.449	1.000	0.025
SKADE	0.449	0.590	0.000	0.620	0.449	1.000	0.120
SWI	0.631	0.834	0.374	0.696	0.553	0.940	0.48
SoluProt	0.657	0.724	0.341	0.672	0.588	0.783	0.321
EPSOL	0.449	0.720	0.02	0.620	0.449	1.000	0.037
NetSolP	0.640	0.742	0.304	0.652	0.576	0.752	0.371
DeepSoluE	0.662	0.737	0.368	0.689	0.587	0.833	0.346
ProG-SOL	0.762	0.856	0.532	0.755	0.703	0.815	0.555

Note: The training data of Protein-Sol, GraphSol, HybridGCN are all from the eSol database[1],

so there is no comparison here. PCC means the Pearson correlation coefficient.

Table S3. Performance of prediction methods on S. cerevisiae dataset.

Methods	ACC	AUC	MCC	F1	Precision	Recall	PCC
CamSol	0.597	0.797	0.348	0.642	0.929	0.491	0.435
Protein-Sol	0.778	0.861	0.536	0.833	0.930	0.755	0.531
PaRSnIP	0.583	0.512	-0.038	0.712	0.725	0.698	0.035
DeepSol-S2	0.444	0.520	0.008	0.500	0.741	0.377	0.077
SKADE	0.292	0.748	0.008	0.105	0.750	0.057	0.293
SWI	0.778	0.820	0.332	0.867	0.776	0.981	0.446
SoluProt	0.750	0.608	0.250	0.845	0.778	0.925	0.179
GraphSol	0.819	0.853	0.561	0.874	0.900	0.849	0.565
EPSOL	0.597	0.556	-0.020	0.724	0.731	0.717	0.112
NetSolP	0.514	0.816	0.346	0.507	1.000	0.340	0.526
DeepSoluE	0.736	0.615	0.000	0.848	0.736	1.000	0.190
HybridGCN	0.819	0.874	0.600	0.869	0.935	0.811	0.603
ProG-SOL	0.847	0.889	0.590	0.899	0.875	0.925	0.611

Table S4. Performance of different graph network models on PSI: Biology dataset (CV5).

GCN type	ACC	AUC	MCC	F1
GraphConv	0.728	0.753	0.365	0.804
GINConv	0.734	0.755	0.369	0.812
GATConv	0.715	0.682	0.317	0.802
ChebConv	0.735	0.747	0.371	0.813
SAGEConv	0.735	0.765	0.400	0.802

Table S5. Performance of different predicted contact probability cutoffs on PSI: Biology dataset (CV5).

Prob. cutoff	ACC	AUC	MCC	F1
0	0.735	0.765	0.400	0.802
0.1	0.733	0.758	0.375	0.808
0.2	0.669	0.532	0.075	0.799
0.3	0.706	0.761	0.390	0.763
0.4	0.734	0.755	0.369	0.812
0.5	0.732	0.747	0.376	0.806
0.6	0.733	0.741	0.383	0.806
0.7	0.731	0.740	0.355	0.812
0.8	0.735	0.736	0.364	0.817
0.9	0.733	0.751	0.366	0.812

Table S6. Performance of repeated training 10 times using the same parameters with ProG-SOL.

Training times	NESG			eSOL				S. cerevisiae				TEM1.1	LGK
	AUC	ACC	F1	AUC	ACC	F1	PCC	AUC	ACC	F1	PCC	AUC	AUC
1	0.76	0.72	0.79	0.82	0.70	0.72	0.51	0.83	0.81	0.88	0.57	0.60	0.60
2	0.78	0.73	0.80	0.84	0.71	0.73	0.55	0.85	0.85	0.90	0.58	0.63	0.57
3	0.75	0.72	0.80	0.83	0.73	0.74	0.51	0.84	0.82	0.89	0.58	0.60	0.62
4	0.77	0.72	0.80	0.82	0.72	0.73	0.51	0.81	0.83	0.90	0.56	0.59	0.57
5	0.78	0.73	0.80	0.85	0.72	0.74	0.56	0.86	0.82	0.88	0.61	0.63	0.60
6	0.78	0.74	0.80	0.85	0.72	0.75	0.56	0.85	0.83	0.89	0.61	0.61	0.58
7	0.78	0.73	0.80	0.85	0.72	0.74	0.56	0.86	0.82	0.88	0.61	0.63	0.60
8	0.77	0.73	0.80	0.82	0.72	0.71	0.51	0.83	0.79	0.88	0.57	0.61	0.56
9	0.78	0.74	0.81	0.86	0.76	0.76	0.56	0.89	0.85	0.90	0.61	0.63	0.57
10	0.77	0.72	0.79	0.84	0.73	0.74	0.54	0.84	0.81	0.87	0.56	0.61	0.56
mean	0.77	0.73	0.80	0.84	0.72	0.74	0.54	0.85	0.82	0.89	0.59	0.61	0.58
std	0.01	0.01	0.01	0.01	0.02	0.01	0.02	0.02	0.02	0.01	0.02	0.01	0.02
ProG-SOL	0.78	0.74	0.81	0.85	0.76	0.76	0.57	0.90	0.85	0.90	0.64	0.63	0.57

Pseudocode of ProG-SOL

using ProtT5-XL-Uniref50 model to get sequence embedding as pre-trained node features
Function get_pretrained_node_feature(sequence)
    pretrained_node_feature = Embedding_Extraction(sequence)
    return pretrained_node_feature
End function

using PSI-BLAST to get PSSM matrix as evolutionary node features
Function get_evol_node_feature(sequence)
    evol_node_feature = Blast_Uniref90(sequence)
    return evol_node_feature
End function

using SPOT-Contact-LM to get contact **probabilities matrix** as edge features
Function get_contact_probs(sequence)
    contact_probs = Contact_Prediction (sequence)
    return contact_probs
End function

generate graph architecture

```
Function graph_generation(pretrained_node_features, evol_node_features, contact_probs, prob_cutoff)
    pair_list = []
    for i from 1 to length(contact_probs):
        for j from 1 to length(contact_probs):
            if contact_probs[i, j]>prob_cutoff then
                pair_list.append(i)
    End for
    graph = build_graph(pair_list)
    graph.node_feat1 = pretrained_node_features[graph.nodes()]
    graph.node_feat2 = evol_node_features[graph.nodes()]
    graph.edge_feat = contact_probs[graph.edges()]
    return graph
End function

# SolubilityClassificationModel
Function ClassificationGraphConvolution(n_conv1, n_conv1, n_fcn, graph)
    node_features_list1 = []
    for i = 1 to n_conv1:
        h1 = SAGEConv(graph, graph.node_feat1, edge_feat)
        h1 = relu(h1)
        h1 = normalization(h1)
        node_features_list1.append(h1)
    node_features1 = Pooling(concatenate(node_features_list1))
    End for

    node_features_list2 = []
    for i = 1 to n_conv2:
        h2 = SAGEConv(graph, graph.node_feat2, edge_feat)
        h2 = relu(h2)
        h2 = normalization(h2)
        node_features_list2.append(h2)
    node_features2 = Pooling(concatenate(node_features_list2))
    End for

    embedding = concatenate(node_features1, node_features2)
    for i = 1 to n_fcn:
        embedding = Linear(embedding)
        embedding = relu(embedding)
        embedding = normalization(embedding)
    End for
    probability = classification_linear(embedding)
    return probability
End function
```

```
#Training
dataset = Dataset(graphs, labels)
loss_function = cross_entropy
optimizer = Adam
for epoch = 1 to num_epochs:
    for batch in dataset:
        inputs, targets = batch
        predictions = model(inputs)
        loss = loss_function(predictions, targets)
        optimizer.zero_grad()
        loss.backward()
        optimizer.step()
```

References

(1) Niwa, T.; Ying, B.-W.; Saito, K.; Jin, W.; Takada, S.; Ueda, T.; Taguchi, H. Bimodal Protein Solubility Distribution Revealed by an Aggregation Analysis of the Entire Ensemble of Escherichia Coli Proteins. *Proc. Natl. Acad. Sci. U.S.A.* **2009**, *106* (11), 4201–4206. https://doi.org/10.1073/pnas.0811922106.
